# Supplementary figures and images for: Distinguishing non severe cases of dengue from COVID-19 in the context of co-epidemics: A cohort study in a SARS-CoV-2 testing center on Reunion island
Source: PLoS Negl Trop Dis. 2021 Apr 26;15(4):e0008879. doi: 10.1371/journal.pntd.0008879 (PMC8102001; doi:10.1371/journal.pntd.0008879)

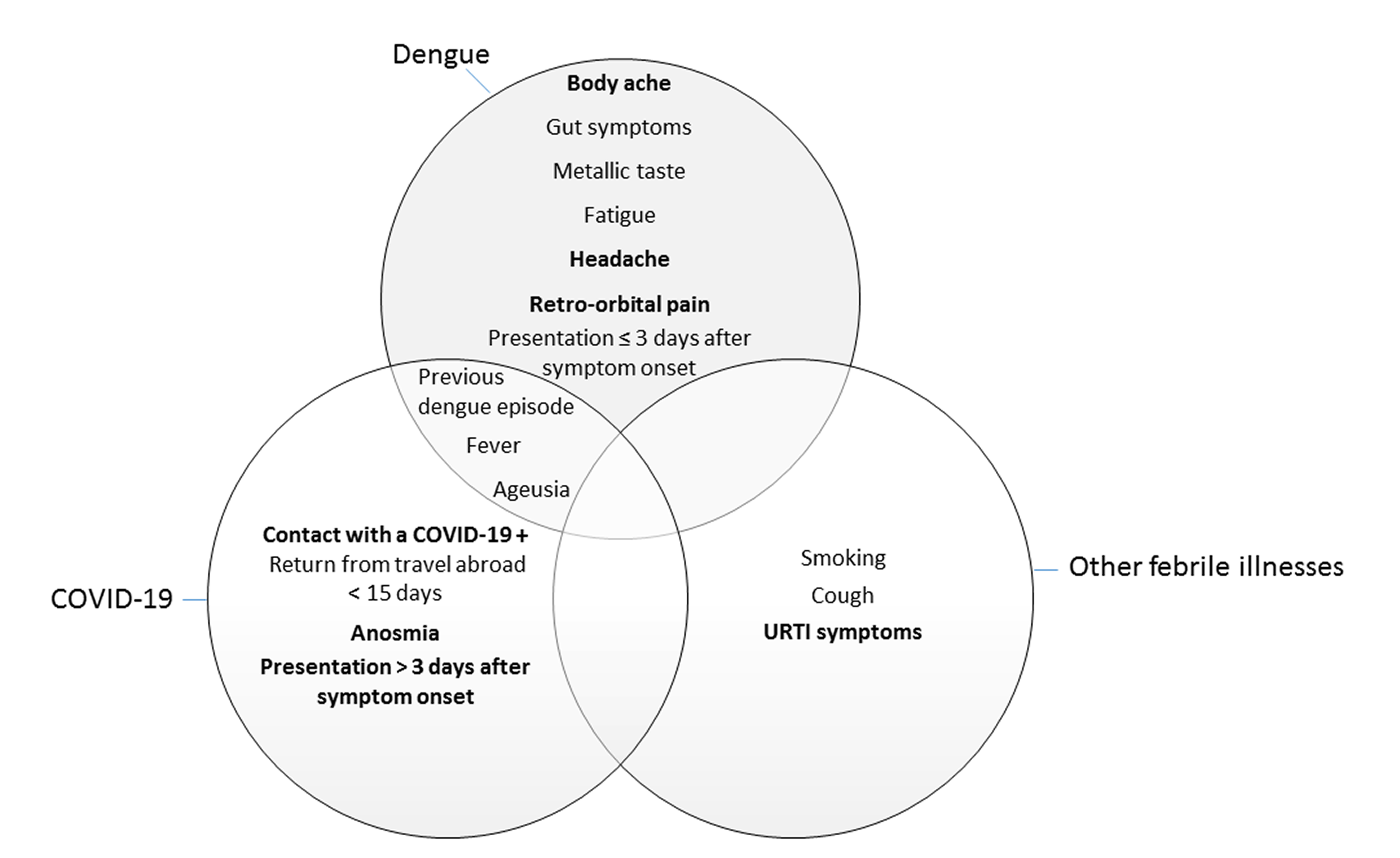

Supplement: S1 Fig — Venn diagram summarizing the predictors for COVID-19, dengue and other febrile illnesses. Predictors for COVID-19 are displayed in the bottom left circle of the Venn diagram, predictors for dengue in the top circle, and predictors for non-COVID-19 non-dengue other febrile illnesses in the bottom right circle. Independent predictors are in bold characters, crude predictors that do not resist to multiple adjustments are in thin characters. (TIF) [file pntd.0008879.s002.tif]
