# Supplementary material for: Distinguishing non severe cases of dengue from COVID-19 in the context of co-epidemics: A cohort study in a SARS-CoV-2 testing center on Reunion island
Source: PLoS Negl Trop Dis. 2021 Apr 26;15(4):e0008879. doi: 10.1371/journal.pntd.0008879 (PMC8102001; doi:10.1371/journal.pntd.0008879)
Supplement: S2 Table — Multinomial logistic regression model with other non COVID-19 non dengue febrile illnesses* taken as controls. In this model, the probability of OFIs cases to be hospitalized was set at 16% (speculated). Data are numbers, weighted cumulative incidence rates (wCIR) expressed as percentages, survey-adjusted odd ratios (s-aOR), 95% confidence intervals (95% CI) and P values for Wald tests. † Current smokers, as compared to never smokers and past smokers. ‡ muscle pain or backache with tightness and/or stiffness. # sore throat, runny nose, nasal congestion, or sneezing. The indicators of performance of the model are unavailable with the svy option in Stata. (DOCX) [file pntd.0008879.s004.docx]

| S2 table. Independent predictors in weighted multivariate analysis (scenario 1) distinguishing COVID-19 and dengue from other febrile illnesses among 972 subjects consulting a COVID-19 screening center during the COVID-19 dengue co-epidemics, Reunion island, Saint-Pierre, March 23-May 10, 2020 | | | | | | | | | | |
| --- | --- | --- | --- | --- | --- | --- | --- | --- | --- | --- |
| Outcomes (versus other febrile illnesses as controls*) | COVID-19 (n = 74) | | | | | Dengue (n = 60) | | | | |
| Predictors | **n** | **wCIR, %** | **s-aOR** | **95% CI** | ***P* value** | **n** | **wCIR, %** | **s-aOR** | **95% CI** | ***P* value** |
| Contact with a COVID-19 positive case | 40 | 17.55 | 8.19 | 3.38 - 19.80 | < 0.001 | 6 | 7.53 | 2.44 | 0.32 - 18.49 | 0.387 |
| Active smoking † | 4 | 1.61 | 0.38 | 0.12 - 1.18 | 0.094 | 12 | 20.92 | 1.93 | 0.21 - 17.67 | 0.562 |
| Cough | 32 | 3.98 | 0.55 | 0.20- 1.45 | 0.225 | 17 | 6.31 | 0.45 | 0.11 - 1.74 | 0.244 |
| Body ache ^‡^ | 29 | 4.40 | 0.82 | 0.33 - 2.04 | 0.669 | 52 | 16.84 | 4.19 | 0.80 - 21.92 | 0.089 |
| Anosmia | 26 | 9.31 | 4.57 | 1.59 - 13.10 | 0.005 | 3 | 10.17 | 1.41 | 0.19 - 10.14 | 0.730 |
| Headache | 28 | 3.45 | 1.09 | 0.41 - 2.83 | 0.864 | 55 | 16.87 | 29.57 | 8.77 - 99.61 | < 0.001 |
| Retro-orbital pain | 1 | 1.05 | 0.36 | 0.02 - 5.75 | 0.468 | 17 | 26.91 | 3.91 | 0.61 - 24.93 | 0.149 |
| URTI symptoms ^#^ | 28 | 3.62 | 0.62 | 0.24 - 1.58 | 0.315 | 20 | 6.82 | 0.45 | 0.09 - 2.20 | 0.323 |
| Presentation > 3 days after symptom onset | 54 | 6.26 | 2.59 | 0.96 - 6.94 | 0.059 | 24 | 8.28 | 0.95 | 0.15 - 5.64 | 0.952 |
| Multinomial logistic regression model with other non COVID-19 non dengue febrile illnesses* taken as controls. In this model, the probability of OFIs cases to be hospitalized was set at 1.5% (observed). Data are numbers, weighted cumulative incidence rates (wCIR) expressed as percentages, survey-adjusted odd ratios (s-aOR), 95% confidence intervals (95% CI) and *P* values for Wald tests. † Current smokers, as compared to never smokers and past smokers ‡ muscle pain or backache with tightness and/or stiffness; ^#^ sore throat, runny nose, nasal congestion, or sneezing. The indicators of performance of the model are unavailable under the *svy* option in Stata. | | | | | | | | | | |
