# Supplementary material for: Distinguishing non severe cases of dengue from COVID-19 in the context of co-epidemics: A cohort study in a SARS-CoV-2 testing center on Reunion island
Source: PLoS Negl Trop Dis. 2021 Apr 26;15(4):e0008879. doi: 10.1371/journal.pntd.0008879 (PMC8102001; doi:10.1371/journal.pntd.0008879)
Supplement: S3 Table — Multinomial logistic regression model with other non COVID-19 non dengue febrile illnesses* taken as controls. In this model, the probability of OFIs cases to be hospitalized was set at 16% (speculated). Data are numbers, weighted cumulative incidence rates (wCIR) expressed as percentages, survey-adjusted odd ratios (s-aOR), 95% confidence intervals (95% CI) and P values for Wald tests. † Current smokers, as compared to never smokers and past smokers. ‡ muscle pain or backache with tightness and/or stiffness. # sore throat, runny nose, nasal congestion, or sneezing. The indicators of performance of the model are unavailable with the svy option in Stata. (DOCX) [file pntd.0008879.s005.docx]

| S3 table. Independent predictors in weighted multivariate analysis (scenario 2) distinguishing COVID-19 and dengue from other febrile illnesses among 972 subjects consulting a COVID-19 screening center during the COVID-19 dengue co-epidemics, Reunion island, Saint-Pierre, March 23-May 10, 2020 | | | | | | | | | | |
| --- | --- | --- | --- | --- | --- | --- | --- | --- | --- | --- |
| Outcomes (versus other febrile illnesses as controls*) | COVID-19 (n = 74) | | | | | Dengue (n = 60) | | | | |
| Predictors | **n** | **wCIR, %** | **s-aOR** | **95% CI** | ***P* value** | **n** | **wCIR, %** | **s-aOR** | **95% CI** | ***P* value** |
| Contact with a COVID-19 positive case | 40 | 19.50 | 4.09 | 2.23 - 7.49 | < 0.001 | 6 | 8.37 | 0.98 | 0.14 - 6.80 | 0.982 |
| Active smoking † | 4 | 2.41 | 0.24 | 0.08 - 0.64 | 0.005 | 12 | 31.36 | 3.49 | 1.11 - 10.99 | 0.032 |
| Cough | 32 | 8.15 | 0.79 | 0.42- 1.48 | 0.468 | 17 | 12.93 | 0.32 | 0.12 - 0.79 | 0.013 |
| Body ache ^‡^ | 29 | 7.68 | 1.08 | 0.58 - 1.99 | 0.812 | 52 | 29.40 | 4.04 | 1.27 - 12.81 | 0.018 |
| Anosmia | 26 | 24.24 | 8.18 | 4.06 - 16.47 | < 0.001 | 3 | 26.49 | 1.66 | 0.41 - 6.59 | 0.473 |
| Headache | 28 | 6.15 | 0.92 | 0.51 - 1.65 | 0.789 | 55 | 30.02 | 33.96 | 11.15 - 103.41 | < 0.001 |
| Retro-orbital pain | 1 | 2.06 | 0.46 | 0.04 - 4.98 | 0.526 | 17 | 52.70 | 3.27 | 0.74 - 14.39 | 0.117 |
| URTI symptoms ^#^ | 28 | 6.83 | 0.49 | 0.27 - 0.87 | 0.016 | 20 | 12.90 | 0.41 | 0.13 - 1.20 | 0.104 |
| Presentation > 3 days after symptom onset | 54 | 11.07 | 1.77 | 1.00 - 3.13 | 0.050 | 24 | 14.66 | 1.34 | 0.44 - 3.98 | 0.602 |
| Multinomial logistic regression model with other non COVID-19 non dengue febrile illnesses* taken as controls. In this model, the probability of OFIs cases to be hospitalized was set at 16% (speculated). Data are numbers, weighted cumulative incidence rates (wCIR) expressed as percentages, survey-adjusted odd ratios (s-aOR), 95% confidence intervals (95% CI) and *P* values for Wald tests. † Current smokers, as compared to never smokers and past smokers ‡ muscle pain or backache with tightness and/or stiffness; ^#^ sore throat, runny nose, nasal congestion, or sneezing. The indicators of performance of the model are unavailable under the *svy* option in Stata. | | | | | | | | | | |
