# Supplementary material for: Distinguishing non severe cases of dengue from COVID-19 in the context of co-epidemics: A cohort study in a SARS-CoV-2 testing center on Reunion island
Source: PLoS Negl Trop Dis. 2021 Apr 26;15(4):e0008879. doi: 10.1371/journal.pntd.0008879 (PMC8102001; doi:10.1371/journal.pntd.0008879)
Supplement: S4 Table — Crude predictors in bivariate analysis distinguishing COVID-19 from dengue from after exclusion of other febrile illnesses among 141 subjects consulting a COVID-19 screening center during the COVID-19 dengue co-epidemics, Reunion island, Saint-Pierre, March 23-May 10, 2020. * Other non COVID-19 non dengue febrile illnesses. Data are numbers, row percentages, and P values for Chi2 or Fisher’s exact tests, unless specified as means, standard deviations, and P values for Mann-Whitney tests. † Current smokers, as compared to never smokers and past smokers. ‡ muscle pain or backache with tightness and/or stiffness. ¶ nausea, vomiting, dyspepsia, eructation or abdominal pain. # sore throat, runny nose, nasal congestion, or sneezing. (DOCX) [file pntd.0008879.s006.docx]

**S4 table. Sensitivity analysis. Crude predictors in bivariate analysis distinguishing COVID-19 from dengue from after exclusion of other febrile illnesses among 141 subjects consulting a COVID-19 screening center during the COVID-19 dengue co-epidemics, Reunion island, Saint-Pierre, March 23-May 10, 2020**

| Outcomes | COVID-19  (n = 80) | | Dengue  (n = 61) | |  |
| --- | --- | --- | --- | --- | --- |
| Predictors | **n** | **(%)** | **n** | **(%)** | ***P* value** |
| Gender |  |  |  |  | 0.258 |
| Male | 33 | 51.6 | 31 | 48.4 |  |
| Female | 47 | 61.0 | 30 | 39.0 |  |
| Age, years |  |  |  |  | < 0.001 |
| 0-30 (Q1) | 28 | 77.8 | 8 | 22.2 |  |
| 31-41 (Q2) | 10 | 27.8 | 26 | 72.2 |  |
| 42-54 (Q3) | 27 | 67.5 | 13 | 32.5 |  |
| 55-94 (Q4) | 15 | 51.7 | 14 | 48.3 |  |
| Contact with a COVID-19 positive case |  |  |  |  | < 0.001 |
| No | 38 | 40.9 | 55 | 59.1 |  |
| Yes | 42 | 87.5 | 6 | 12.5 |  |
| Return from travel abroad < 15 days |  |  |  |  | < 0.001 |
| No | 37 | 40.2 | 55 | 59.8 |  |
| Yes | 42 | 87.5 | 6 | 12.5 |  |
| Previous dengue episode |  |  |  |  | 0.174 |
| No | 73 | 58.4 | 52 | 41.6 |  |
| Yes | 6 | 40.0 | 9 | 60.0 |  |
| Active smoking ^†^ |  |  |  |  | 0.008 |
| No | 73 | 59.8 | 49 | 40.2 |  |
| Yes | 4 | 25.0 | 12 | 75.0 |  |
| Fever |  |  |  |  | < 0.001 |
| No | 35 | 94.6 | 2 | 5.4 |  |
| Yes | 45 | 43.3 | 59 | 56.7 |  |
| Duration of fever (days), µ ± sd | 3.43 | 3.35 | 3.03 | 2.88 | 0.892 |
| Cough |  |  |  |  | 0.037 |
| No | 44 | 50.0 | 44 | 50.0 |  |
| Yes | 36 | 67.9 | 17 | 32.1 |  |
| Duration of cough (days), µ ± sd | 2.14 | 12.84 | 5.79 | 7.98 | 0.958 |
| Dyspnea/Shortness of breath |  |  |  |  | 0.443 |
| No | 67 | 58.3 | 48 | 41.7 |  |
| Yes | 13 | 50.0 | 13 | 50.0 |  |
| Duration of dyspnea (days), µ ± sd | 5.44 | 8.43 | 7.75 | 5.25 | 0.771 |
| Body ache ^‡^ |  |  |  |  | < 0.001 |
| No | 48 | 84.2 | 9 | 15.8 |  |
| Yes | 32 | 38.1 | 52 | 61.9 |  |
| Duration of pain (days), µ ± sd | 4.34 | 5.49 | 2.90 | 2.72 | 0.399 |
| Diarrhea |  |  |  |  | 0.732 |
| No | 61 | 56.0 | 48 | 44.0 |  |
| Yes | 19 | 59.4 | 13 | 40.6 |  |
| Duration of liquid stools (days), µ ± sd | 4.50 | 3.79 | 2.25 | 3.14 | 0.087 |
| Gut symptoms ^¶^ |  |  |  |  | 0.003 |
| No | 76 | 61.3 | 48 | 38.7 |  |
| Yes | 4 | 23.5 | 13 | 76.5 |  |
| Ageusia |  |  |  |  | 0.075 |
| No | 55 | 52.4 | 50 | 47.6 |  |
| Yes | 25 | 69.4 | 11 | 30.6 |  |
| Duration of ageusia (days), µ ± sd | 4.73 | 3.32 | 3.25 | 2.01 | 0.271 |
| To be continued… |  |  |  |  |  |

| Anosmia |  |  |  |  | < 0.001 |
| --- | --- | --- | --- | --- | --- |
| No | 52 | 47.3 | 58 | 52.7 |  |
| Yes | 28 | 90.3 | 3 | 9.7 |  |
| Duration of anosmia (days), µ ± sd | 4.22 | 3.59 | 1.00 | 1.00 | 0.093 |
| Fatigue |  |  |  |  | < 0.001 |
| No | 42 | 77.8 | 12 | 22.2 |  |
| Yes | 38 | 43.7 | 49 | 56.3 |  |
| Duration of fatigue (days), µ ± sd | 6.48 | 5.75 | 3.44 | 3.00 | 0.009 |
| Headache |  |  |  |  | < 0.001 |
| No | 49 | 90.7 | 5 | 9.3 |  |
| Yes | 31 | 35.6 | 56 | 64.4 |  |
| Duration of headache (days), µ ± sd | 4.69 | 5.61 | 3.02 | 2.74 | 0.216 |
| Retro-orbital pain |  |  |  |  | < 0.001 |
| No | 79 | 64.2 | 44 | 35.8 |  |
| Yes | 1 | 5.6 | 17 | 94.4 |  |
| URTI symptoms ^#^ |  |  |  |  | 0.465 |
| No | 49 | 54.4 | 41 | 45.6 |  |
| Yes | 31 | 60.8 | 20 | 39.2 |  |
| Duration of rhinorrhea (days), µ ± sd | 5.33 | 3.69 | 2.10 | 0.91 | 0.008 |
| Presentation > 3 days after symptom onset |  |  |  |  | < 0.001 |
| No | 23 | 39.0 | 36 | 61.0 |  |
| Yes | 54 | 69.2 | 24 | 30.8 |  |
| Time elapsed since symptom onset (days), µ ± sd | 7.54 | 6.50 | 4.18 | 4.57 | < 0.001 |
| * Other non COVID-19 non dengue febrile illnesses. Data are numbers, row percentages, and *P* values for Chi2 or Fisher’s exact tests, unless specified as means, standard deviations, and *P* values for Mann-Whitney tests. † Current smokers, as compared to never smokers and past smokers. ^‡^ muscle pain or backache with tightness and/or stiffness. ^¶^ nausea, vomiting, dyspepsia, eructation or abdominal pain. ^#^ sore throat, runny nose, nasal congestion, or sneezing. | | | | | |
