# Supplementary material for: Distinguishing non severe cases of dengue from COVID-19 in the context of co-epidemics: A cohort study in a SARS-CoV-2 testing center on Reunion island
Source: PLoS Negl Trop Dis. 2021 Apr 26;15(4):e0008879. doi: 10.1371/journal.pntd.0008879 (PMC8102001; doi:10.1371/journal.pntd.0008879)
Supplement: Attachment — Submitted filename: Response to reviewers.pdf [file pntd.0008879.s010.pdf]

PLOS Neglected Tropical Diseases
****
Reviewer's Responses to Questions

Methods

Reviewer #1: -Are the objectives of the study clearly articulated with a clear testable hypothesis stated?
Yes
-Is the study design appropriate to address the stated objectives? Yes
-Is the population clearly described and appropriate for the hypothesis being tested? Yes
-Is the sample size sufficient to ensure adequate power to address the hypothesis being tested? Yes
-Were correct statistical analysis used to support conclusions? Yes
-Are there concerns about ethical or regulatory requirements being met? Yes
We are grateful of the reviewer #1 for his appreciations.

Reviewer #2: small number recruited, and hence analyses are not reliable.

We acknowledge the number of COVID-19 and dengue cases may seem small but these are data collected in the real situation given the diagnostic means allocated to the care of suspected COVID-19 patients at that time. For sure, Reunion Island has benefited early from the national lockdown, three days after the first case was diagnosed, with a consequence of very low COVID-19 incidence, which accounts for the small sample size. This being said, we added page 11, lines 231 to 235 two sentences to address this comment:

"Together with the abovementioned sources of bias, a lack of power might have reduced the capability to shed light on other discriminating factors. However, we believe that this study faithfully reflects the real epidemiological situation on Reunion island at that time, given diagnostic practices and means that were commonly used in this uncertainty era, which is unlikely to have biased the overall sense of our results."

Reviewer #3: The rapid assay test used to diagnose dengue virus infection was the OnSiteTM Duo dengue Ag-IgG-IgM rapid diagnostic test based on the NS1 antigen. If test results were negative a DENV RT-PCR or a dengue serology were performed. Can the authors comment on the sensitivity and specificity of the point of care assay? This is critical to make sure dengue cases were not misclassified or over diagnosed through an assay that may carry a high false positive rate.

According to the manufacturer (CTK Biotech, San Diego, CA, USA), the diagnostic performance of the OnSite™ Duo dengue Ag-IgG-IgM RDT is as follows:

NS1 antigen test: relative sensitivity: 100% (95%CI: 96.8-100%),
relative specificity: 99.6% (95%CI: 97.5-97.8%),
overall agreement (well-classified): 99.7% (95%CI: 98.4-99.9%).

IgG test: relative sensitivity: 97.3% (95%CI: 86.2-100%),
relative specificity: 99.3% (95%CI: 97.5-99.8%),
overall agreement (well-classified): 99.1% (95%CI: 97.3-99.7%).

IgM test: relative sensitivity: 96.9% (95%CI: 84.3-99.4%),
relative specificity: 98.9% (95%CI: 96.9-99.6%),
overall agreement (well-classified): 98.7% (95%CI: 96.7-99.5%).

Cross-reactivity: No false positive with Chikungunya, CMV, HAV, HBV, HCV, HIV, hCG, H. pylori, TB*, T. Gondii*, typhoid, rubella, ANA and HAMA.

According to the recent DENG-RUN study that was conducted 1 year earlier, a retrospective cohort study of dengue patients on Reunion island, the diagnostic performance for the NS1 antigen alone among 197 patients suspected for dengue was: sensitivity 47.7% (95%CI 35.1-60.5%), specificity 58.1% (95%CI 49.1-66.8%) and overall agreement (well-classified) 54.6% (95%CI 47.3-61.8%).

Given the abovementioned data and the epidemiologic situation of dengue on Reunion island (recent progression to endemicity with multiple serotype circulation), we believe the matter of the POC test of dengue, is more a question of false negatives than a question of false positives. With respect to our results, we believe the false negative rate of SARS-CoV-2 may have conducted to classify coinfections with cough or upper respiratory tract infections (URTI) symptoms as dengue rather than as coinfections that should have been ruled out.

Nevertheless, to address this comment we added page 11, lines 224 to 225 ", rather than from the false positive rates." to the sentence "*Second, our results might also be affected by a misclassification bias, which may arise both from the poor sensitivity of SARS-CoV-2 RT-PCR and rapid NS1 antigen.*"

The methods section does not detail any characteristics of participants diagnosed with OFIs. Were other diagnoses entertained, for example chikungunya, or TB or leptospirosis or strep throat for example?

Other diagnoses were entertained on clinical indication when needed, but this study really reflects the real situation of an observational study during the COVID-19 pandemic within a SARS-CoV-2 testing center. This is not an academic clinical research allowing a full differential diagnosis work-up. SARS-CoV-2 testing centers welcome patients with mild to moderate illness. Patients suffering severe symptoms or for which the disease or comorbidities had the potential for life-threatening course were directed towards hospitalization units. Patients not requiring hospitalization were discharged home and benefited an ambulatory follow-up ensured by general practitioners.

There should be an explanation of how the control group was defined. Were these patients who had a febrile illness and tested negative for COVID-19 and dengue?

Other febrile illnesses regroup all the subjects tested negative for SARS-CoV-2 and considered negative for dengue, either clinically, virologically or serologically. This has been specified in the Method Section Page 6, lines 136 to 138. "*Other febrile illnesses (OFIs) were defined as patients tested negative for SARS-CoV-2 and further considered as unrelated to dengue, either clinically, virologically, or serologically.*"

Were OFIs diseases that were not associated with any specific diagnosis?

Yes. Given that the SARS-CoV-2 testing center welcomed patients with mild to moderate illnesses, large majority of our cohort was discharged home without any diagnosis, which reflects yet the daily clinical practice for the majority of mild to moderate spontaneously recovering diseases all over the world. We provide below the full list of primary diagnoses retained for the 16 cases of OFIs that were hospitalized and investigated.

cases	Primary diagnosis	Comment	cases	Primary diagnosis	Comment
1	URTI	Influenza-	9	Bacterial pneumonia	
2	Pleural effusion and pericarditis	BK-, dengue-, long COVID ?	10	Bacterial pneumonia	
3	URTI	Influenza-, RSV-	11	URTI ?	FT21 RV -
4	Virosis (airways, gut)	TDM-, dengue-	12	LRTI	Influenza-, RSV
5	Pleural effusion	Chest cancer, BK-	13	Pleural effusion	TDM-. FT21 RV-
6	Pharyngitis, URTI	Influenza-, RSV+	14	Influenza-like ilness, dengue-like syndrome	Dengue-
7	URTI	Influenza	15	Fever, dehydration	Diabetes, dengue-
8	Bacterial pneumonia		16	Asthma, LRTI	FT21 RV-, dengue-

In addition to the abovementioned, leptospirosis was researched in cases of pneumonia with dyspnea.

The methods section states that patients with co-infections were excluded. Can this statement be clarified? Does that mean participants who had either COVID-19 or dengue and some other condition?

The statement has been clarified page 6, line 135. It was about COVID-19-dengue coinfections.

If so how was the other disease diagnosed? Is there an estimate of how many participants were not enrolled because of co-infections?

During the study period, only two cases of COVID-19-dengue co-infections were recorded. The first was tested SARS-CoV-2 positive in the emergency department, discharged home and readmitted for dengue seven days after. He was hospitalized without being assessed in the SARS-CoV-2 and published in PNTD (Verduyn M et al, PLoS Negl Trop Dis 2020). The second was a case included in our cohort, he was tested positive for SARS-CoV-2 in the UDACS, discharged home, recovered, then was readmitted for dengue weeks later. Given the asynchronicity between the two infections in this patient, he was kept in our analysis addressing the predictors at clinical presentation.

Results

Reviewer #1: -Does the analysis presented match the analysis plan? Yes
-Are the results clearly and completely presented? Yes
-Are the figures (Tables, Images) of sufficient quality for clarity? Yes

We are grateful of the reviewer #1 for his appreciations.

Reviewer #2: (No Response)

Reviewer #3: What diseases were representative of OFIs?

Like almost everywhere else all over the world, in the setting of observational research, OFIS that are most often mild to moderate spontaneously recovering diseases, remained undiagnosed. We provide in the table below the primary diagnoses for OFI cases that were hospitalized and investigated.

cases	Primary diagnosis	Comment	cases	Primary diagnosis	Comment
1	URTI	Influenza-	9	Bacterial pneumonia	
02	Pleural effusion and pericarditis	BK-, dengue-, long COVID ?	10	Bacterial pneumonia	
3	URTI	Influenza-, RSV-	11	URTI ?	FT21 RV -
4	Virosis (airways, gut)	TDM-, dengue-	12	LRTI	Influenza-, RSV
5	Pleural effusion	Chest cancer, BK-	13	Pleural effusion	TDM-. FT21 RV-
6	Pharyngitis, URTI	Influenza-, RSV+	14	Influenza-like ilness, dengue-like syndrome	Dengue-
7	URTI	Influenza	15	Fever, dehydration	Diabetes, dengue-
8	Bacterial pneumonia		16	Asthma, LRTI	FT21 RV-, dengue-

There has been an overall decrease in circulation of influenza and other viral respiratory pathogens during the COVID-19 pandemic, so it is unusual that respiratory symptoms were predominantly associated with OFIs since there was an overall decline in the circulation of viral respiratory pathogens, particularly those that cause fever such as influenza.

We agree with this statement from a theoretical point of view. Our study was performed between March, 23 and May 10 before the influenza season (May to October in the Southern hemisphere). We believe nevertheless the progression of COVID-19 was too low at that time (incidence 41 cases per 100.000 inhabitants) to affect the circulation of other respiratory viruses by competition. Indeed, concomitant to the introduction of SARS-CoV-2on the island, our lab identified a circulation of NIRVs (non-influenza respiratory viruses), especially rhinoviruses, using a fast-track multiplex RT-PCR targeting 21 common respiratory viruses. Among the OFI cases that were included in our study, we identified only 1 case of NIRV infection, which was one RSV (respiratory syncytial virus) infection (cf. Table). The explanation may be otherwise (cf. see infra).

It is also unusual that respiratory symptoms were protective factors against a COVID-19 diagnosis since respiratory symptoms are heavily associated with COVID-19 (sore throat, runny nose, nasal congestion or sneezing cited in the manuscript). Is it possible that there were false negative results from RT-PCR for SARS CoV-2?

We agree with this statement. Nevertheless, we must remain factual regarding statistics in observational research. The fact that URTI symptoms were protective for COVID-19 may indeed reflect misclassification in diagnosis due to false negatives (lack of sensitivity of SARS-CoV-2 RT-PCR test), unmeasured confounding factors (that may skew inversely the association), or a circulation of NIRVs (that makes the association positive). Whatever the case situation, we feel honest to remain factual and not to comment further, as none of these possibilities can be investigated more thoroughly. That does not mean that results are unreliable, as stated without argument by another reviewer. This just means that there is uncertainty. Uncertainty has been accounted and presented as a limitation in the discussion.

Were the nasal swabs self-collected?

Nasal swabs were collected by a nurse following the method that has been described in the video published on the New England Journal of Medicine (Marty FM, Chen K, Verrill KA. Et al, NEJM 2020). We specified "*by a skilled nurse*", page 6, line 125 and within the Methodological appendix (Supp-1).

What was the expected sensitivity and specificity of the SARS CoV-2 RT PCR assay used?

In the absence of clear-cut gold standard, we may assume that the expected sensitivity and specificity of the SARS-CoV-2 RT PCR assay were probably within the expected ranges alleged in the literature for nasopharyngeal swabs (2% to 29% false negatives rate, ~5% false positives rate). In our center, like everywhere else, the diagnosis of COVID-19 was based on clinical adjudication, which relied on both clinical evaluation (history, contact with COVID-19+ case, physical examination, chest X ray± chest CT scan, and biology) and RT-PCR testing (repeated when possible).

What do the authors propose as a potential explanation for these findings?

Which findings? The fact that patients with URTI symptoms were tested negative? Like reminded in the BMJ (Watson J & Brush JE, BMJ 2020, we agree that a single negative COVID-19 test should not be used as a rule-out in patients with strongly suggestive symptoms. With imperfect tests, a negative result means only that a person is less likely to be infected.

It is also unusual that cough would be so prevalent in dengue patients. As alluded to in the discussion, misclassification of participants may have been considerable.

We fully agree with this statement, though misclassification cannot be quantified. In addition, we must also confess the possibility of a reporting bias. Also, while misclassification is theoretical and agreed within the scientific community due to the intrinsic qualities of SARS-CoV-2 testing, over-reporting of

URTI symptoms and cough in fear of COVID-19 could not be ruled out at that time, when the first pandemic wave began. To address this other potential source of bias, we reanalyzed our data (cf. reporting_bias.txt file, supp-3) and added page 11, lines 226 to 230, the following paragraph:

"Third, given the fear of COVID-19 at that time, we cannot rule out the possibility of a reporting bias, as some patients may have declared URTI symptoms or cough in excess just to be tested for SARS-CoV-2. Consistent with this, are the relatively high percentages of cough and URTI symptoms among dengue cases as well as the totality of anxiety cases upon testing observed within the OFI group (data not shown), for instances."

Nonetheless, 100% of the dry cough episodes objectified upon testing occurred among patients that had declared cough as a symptom, which tempers false declarations and over-reporting, airway reactiveness being triggered more by infection (common cold and rhinoviruses for instance) than anxiety (Svensson C et al, Am J Rhinol 1998;Mygind N, Monaldi Arch Chest Dis 2000).

Why would smoking be associated with a lower likelihood of COVID-19? What would explain such an observation?

Like for the lower likelihood of COVID-19 with URTI symptoms, the lower likelihood of COVID-19 among active smokers may proceed from several mechanisms: under-reporting of smokers in COVID-19 patients (leading to misclassification by differential classification bias), unmeasured confounding factors (that may skew positively the protective association), or a real protective effect of active smoking (so far undocumented). Recent evidence suggests that smokers express more ACE2 entry receptor in bronchial and alveolar epithelia, which should increase the risk of infection (Zhang H et al, Am J Resp Crit Care Med 2020; Liu A et al, J Pathol 2021). In accordance with these articles, we temper our comment adding two sentences, page 13, lines 269 to 273.

"This finding seems paradoxical given recent evidence shows that Angiotensin-converting enzyme 2 (ACE2), the SARS-CoV-2 entry receptor, is overexpressed in smoker's bronchial and alveolar epithelia, which should increase the risk of infection [16-18]. Whether this finding results from abovementioned misclassification or reporting bias deserves further studies."

It was thought in the early days of the pandemic that smoking would make pulmonary disease worse.

We disagree with this statement. The data regarding the effects of smoking among COVID-19 patients are indeed very heterogeneous, both for disease incidence and disease progression, which has led the community to discuss an apparent so-called smoker's paradox in COVID-19 (Usman MS et al, BMJ Evidence Based Med 2020). Given these data came from observational research, there is no possibility to conclude with strong confidence owing to sources of biases. This being said making pulmonary disease worse is about disease prognosis (anti-inflammatory role of nicotine?), which is downstream to protection from infection (disease acquisition), and there are many paradoxical examples in medicine that show both protection from disease and poor outcome once diseased. For example, maternal smoking may accelerate lung maturation in preterm neonates and prevent hyaline membrane disease, but respiratory distress syndrome may be more severe in neonates born from smokers due to incomplete lung development.

One potential explanation is the small sample size of smokers, in table S1 there are only 4 smokers in the COVID-19 category and 12 in the dengue category.

We fully agree with this statement that small sample sizes may lead to inconsistent findings. That is why rather than take a stance on controversial data, we have preferred to show the inconsistency of our findings (through comparisons in un-weighted and weighted analyses, cf. new table S2 and table S3) and remain factual. We are confident that meta-analysis of observational data will solve this issue. This being said, as the numbers of active smokers among OFI and dengue subjects were within the usual reported

range on Reunion island, we have no argument to argue a selective under-reporting of active smoking in COVID-19 patients.

COVID-19 symptoms vary by age. What was the age of study participants, and how did disease presentation vary by age?

We thank the reviewer for this comment. To address these two questions, we provide two ppt slides presenting the age distribution for all participants according to each outcome group (OFIs, COVID-19 and dengue) and a text file (symptoms_by_age.txt, supp-3) presenting a stratified analysis by age quartiles within each outcome group. With an exception, this shows no interaction between age and disease presentation (risk factors, symptoms) within each outcome group. Therefore, from our dataset, we cannot argue the assertion that symptoms vary by age.

It appears that age distribution was not significant for COVID-19 cases but it was significant in dengue cases, with the younger age group being more protected against dengue. There is no comment about this finding in the results or discussion but it would be worth providing a potential explanation for this finding.

We thank the reviewer for this comment. We think it is difficult to interpret the effect of age on each disease presentations and that there is no strong lesson to draw from such inconsistent observation. This would not be the case if the age distribution followed a linear relationship or was U-shaped, which could allow then an immunopathologic interpretation. This is not the case in our study. Given the absence of interaction between age and disease presentation and the inconsistency of the finding, we prefer removing age from our model not to be incentive and lead to wrong interpretations. In our former analysis, we had left the age in our model because a quick comparison of the age in quartiles between dengue and COVID-19 patients (excluding OFIs) had shown a highly significant difference (Table S2). Given the heterogeneity of epidemiologic situations regarding age, we now believe that it is preferable to remove this factor to ensure a better external validity (possibility to replicate our findings). Indeed, the effect of age on dengue presentation may be different according to different contexts, both for children and the elderly populations according to how long dengue is endemic. Alternatively, the effect of age on COVID-19 presentation may be different according to which population travel (young workers, elder retired).

Cough was a protective factor against dengue in table 1, but not necessarily associated with COVID-19 which is unusual. Also unusual that URTI symptoms would be a protective risk factor for COVID-19, it was also protective for dengue but that would be expected. This is highly suggestive of potential misclassification, with potentially COVID-19 patients being transferred to the OFIs group because of a negative test result, which would explain the unusual high frequency of URTIs in the control group.

We agree with these comments already noted by the reviewer (cf. read above, pages 3-4). Misclassification has been advocated as possible explanation, page 11, lines 223 to 225, as in the first version of manuscript. This cannot be opposed to us as it is the proper of observational research and it was put ahead as a possible limitation.

The frequencies of the clinical/ demographic parameters are not reported for OFIs. They should be displayed in a table along with the other two illnesses.

We thank the reviewer for this comment and now provide a table (Table 1) comparing in bivariable analysis the three outcome groups on numerous characteristics.

Conclusions

Reviewer #1:

-Are the conclusions supported by the data presented? Yes
-Are the limitations of analysis clearly described? Yes
-Do the authors discuss how these data can be helpful to advance our understanding of the topic under study? Yes
-Is public health relevance addressed? Yes

We are grateful of the reviewer #1 for his appreciations. We also explored reporting bias as another possibility of limitation and added it to the list. We hope this will rend our manuscript more insightful regarding the difficulties to conduct observational research in the context of pandemic.

Reviewer #2: Dengue versus COVID-19 at presentation are so different, and the conclusion in the abstract and manuscript is misleading. "these data suggest that non-severe dengue may be more symptomatic than COVID-19 in a co-epidemic setting with higher dengue attack rates. At clinical presentation, basic clinical and epidemiological indicators may help to distinguish COVID-19 or dengue from each other and other febrile illnesses."

We thank the reviewer for reminding us the obvious. However, at the time when we submitted this article, there was no clinical data published from an observational study comparing COVID-19 and dengue presentations, and evidence could not be based otherwise that provided by experts. Our data are issued from a very special context, a SARS-CoV-2 testing center located in a low incidence setting for COVID-19 and an epidemic setting for dengue, which welcomed mild to moderate cases of both diseases, in the middle of circulation of non-influenza respiratory viruses. We think that all that has been written from contexts of huge first pandemic waves of Covid-19 can be forgotten. For instance, in the absence of huge circulation of SARS-Co-V-2, viral competition extinguishing the circulation of other respiratory viruses cannot take place.

Dengue presents in younger persons. COVID-19 affects the bulk of all age groups, but severe cases mainly only occur in older people. COVID-19 is usually mild in the first days and then can progress to severe disease usually around day 10 of illness with rapid progression to death if no access to oxygen and more supportive care is available.

We agree with these assertions but in our context, it has been observed within the hospital setting, not in the SARS-CoV-2 testing center which resembles an outpatient clinic with mild to moderate illnesses.

It would also be misleading to rely on clinical and epidemiological indicators. The key message should be that all cases should have a diagnostic work-up for COVID-19, as underdiagnosing COVID-19 could propagate further transmission.

We agree with this statement. However, it is not helpful at all both for the clinician in the field who must sometimes rely on inaccurate diagnostic tools, and the researcher. As a result, we would prefer to be optimistic and base our conclusions on our findings.

The consequences of unmitigated transmission is huge. Misdiagnosing dengue does not have so many repercussions as the vast majority of cases improve spontaneously, and there is no exponential growht in onward transmission.

We agree with the first assertion. The second is erroneous. Exponential growth can be seen with vector-borne diseases. We, researchers from Réunion island know something about it (chikungunya 2006 epidemic) and believe that we can be trusted for claiming that so.

Reviewer #3:

A study limitation is the absence of an asymptomatic tested population. Since both dengue and COVID-19 have high asymptomatic infection rates, it would have been of interest to explore positivity rates for both conditions in this setting, as a research opportunity. It is understandable, however, that this population was not evaluated given that the main hypothesis focused on clinical parameters associated with each condition.

We agree with this comment. As mentioned by the reviewer, we focused on clinical parameters and thereby excluded asymptomatic subjects. We think asymptomatic tested populations are better investigated in the setting of contact tracing and seroepidemiological studies, not in the setting of hospital-based studies.

Editorial and Data Presentation Modifications?

Reviewer #1: Line 191: COVID+ should be corrected to COVID-19+

Done. Page 10, now line 201

Line 246 ? OFis should be corrected to OFIs

Done. Page 10, now lines 204 and 209, as well as page 13, line 267.

Reviewer #2:

Reviewer #3: More information about OFIs is needed in the manuscript.

We thank the reviewer for this comment. More information has been given for OFIs in terms of clinical presentation, duration of symptoms and disease outcome in the new table 1. Additional information is given in terms of diagnosis for OFIs cades that were hospitalized. We regret that in the context of a SARS-CoV-2 testing center which resembles to an outpatient clinic treating mild to moderate clinical forms, more investigations could not have been made, but this is the reality of most mild illnesses that recover and quickly and spontaneously.

The issue of potential misclassification of COVID-19 cases as OFIs should be further addressed, particularly when there are findings that are not compatible with the clinical conditions represented.

We agree with the first assertion and have provided over-reporting of URTI syndrome and cough as a possible explanation (cf. reporting_bias.txt file, supp-3) for misclassification (differential classification bias) but disagree that our findings are not compatible with the clinical conditions presented. Our findings are issued from a very special context, so far original, that report similitudes in mild disease presentation. This is not the hospital setting where presentations fulfil their full potential for specificities and differences.

The degree of fever could potentially differentiate between the two conditions, the analysis does not look at high versus low fever.

We agree with this statement, but fever should be better investigated in the setting of a hospitalization unit, not in that of an outpatient clinical. Nevertheless, we have provided in the new table 1, data on fever frequency, magnitude, and duration.

Stratification of clinical findings by age would also be something of interest, particularly for COVID as the disease differs across age groups.

We thank the reviewer for this comment. As abovementioned Page 6, line 248 of this document, we have stratified each symptom by age group according to each outcome and provide text file (symptoms_by_age.txt) to show it. The result is that there is no interaction between symptoms and age. With an exception, the disease does not differ across age groups for each outcome group.

Some comment about dengue prevalence in certain age groups is worth including, as younger age groups seemed less likely to have dengue.

As abovementioned page 5, line 260 of this document, we believe the effect of age is fortuitous and inconsistent. This would not be the case if the age distributions followed a linear relationship or was U-shaped, which could allow then an immunopathologic interpretation. In our study, we cannot rule out a selection bias, children being rather oriented towards pediatric emergencies rather than the SARS-CoV-2 testing center. Moreover, we strongly believe that such an issue should be addressed by a population-bases seroepidemiological study, less prone to selection bias than a hospital-based study.

Indeed, there is an immunopathological reason that could support higher proportions of inapparent cases (asymptomatic and pauci-symptomatic undetected) in children than in adults when endemicity is recent, which is the case of Reunion island, where annual epidemics date back from 2015 and cocirculation of multiple serotypes dates back from 2019. A minimum of eight years has been advanced foro bserving higher risks of infection and subsequent disease (Tsang TK et al, Nat Com 2019).

Summary and General Comments

Use this section to provide overall comments, discuss strengths/weaknesses of the study, novelty, significance, general execution and scholarship. You may also include additional comments for the author, including concerns about dual publication, research ethics, or publication ethics. If requesting major revision, please articulate the new experiments that are needed.

Reviewer #1: As Severe Acute Respiratory Syndrome Coronavirus-2 (SARS-CoV-2) is spreading globally, worldwide growing concerns about the risks of overlapping epidemics and co-infections with emergent viruses, especially with arboviruses, have being intensifying. Here, the authors designated a retrospective cohort study aimed at identifying the clinical and epidemiological profiles of SARS-CoV-2 and DENV infections to guide their management and mitigate the impact of COVID-19 pandemic surge locally. The paper is generally interesting to the scientific community and infectious disease experts in particular. A strength of this study is to prepare the healthcare system to better and rapid distinguish between both SARS-CoV-2 or DENV infections, avoiding delays in treatment and preventable deaths.

We are indebted of the reviewer for this positive comment.

Reviewer #2: Dengue versus COVID-19 at presentation are so different, and the conclusion in the abstract and manuscript is misleading. "these data suggest that non-severe dengue may be more symptomatic than COVID-19 in a co-epidemic setting with higher dengue attack rates. At clinical presentation, eight basic clinical and epidemiological indicators may help to distinguish COVID-19 or dengue from each other and other febrile illnesses."

Dengue presents in younger persons. COVID-19 affects the bulk of all age groups, but severe cases mainly only occur in older people. COVID-19 is usually mild in the first days and then can progress to severe disease usually around day 10 of illness with rapid progression to death if no access to oxygen and more supportive care is available.

It would also be misleading to rely on clinical and epidemiological indicators. The key message should be that all cases should have a diagnostic work-up for COVID-19, as underdiagnosing COVID-19 could propagate further transmission. The consequences of unmitigated transmission is huge. Misdiagnosing dengue does not have so many repercussions as the vast majority of cases improve spontaneously, and there is no exponential growht in onward transmission.

We agree with the reviewer but prefer relying our conclusions on our results rather than stating the obvious. It is up to us to propose a modest contribution to knowledge based on original findings issued from a particular context rather than emphasizing what is already known. A full diagnostic work-up would be possible in the setting of hospitalization but is not suitable in an outpatient clinic confronted with mild to moderate illness quickly reversible nor in the framework of an observational research.

Reviewer #3: The manuscript proposes an interesting analysis of clinical relevance, which is to find demographic/ clinical parameters that may help differentiate between dengue infection and COVID-19 disease in a setting highly endemic for arboviral illnesses. A comparison group of other febrile illnesses is included in the analysis but this group needs to be better characterized, with definitions and more data provided about potential diagnoses in this control population. More data about clinical findings in the OFI population should also be provided in the tables. The authors should try to further address the issue of potential misclassification of diagnoses, since some of the findings do not appear to be biologically plausible, such as respiratory symptoms being protective against a COVID-19 diagnosis for example. This merits further investigation. Some of the other results are highly plausible. The degree of fever could potentially differentiate between the two conditions, the analysis does not look at high versus low fever. Stratification of clinical findings by age would be something of interest, particularly for COVID as the disease differs across age groups.

We have provided more data for characterizing the control group of other febrile illnesses in the new Table 1. Nevertheless, the mild to moderate disease presentation observed and their reversible character, as well as the framework of an observational research, do not allow us to be more exhaustive in the diagnostic work-up, and definitely to better characterize the OFI control group.

Figure Files:
While revising your submission, please upload your figure files to the Preflight Analysis and Conversion Engine (PACE) digital diagnostic tool, https://pacev2.apexcovantage.com. PACE helps ensure that figures meet PLOS requirements. To use PACE, you must first register as a user. Then, login and navigate to the UPLOAD tab, where you will find detailed instructions on how to use the tool. If you encounter any issues or have any questions when using PACE, please email us at figures@plos.org.

We will do this.

Data Requirements:
Please note that, as a condition of publication, PLOS' data policy requires that you make available all data used to draw the conclusions outlined in your manuscript. Data must be deposited in an appropriate

repository, included within the body of the manuscript, or uploaded as supporting information. This includes all numerical values that were used to generate graphs, histograms etc.. For an example see here: http://www.plosbiology.org/article/info%3Adoi%2F10.1371%2Fjournal.pbio.1001908#s5.
We agree to make our data available in a data repository

Reproducibility:
To enhance the reproducibility of your results, PLOS recommends that you deposit laboratory protocols in protocols.io, where a protocol can be assigned its own identifier (DOI) such that it can be cited independently in the future. For instructions see https://journals.plos.org/plosntds/s/submission-guidelines#loc-methods

We have no protocol for this retrospective observational research.

In compliance with data protection regulations, you may request that we remove your personal registration details at any time. (Use the following URL: https://www.editorialmanager.com/pntd/login.asp?a=r). Please contact the publication office if you have any questions.
